# Hypersensitivity Pneumonitis Due to Living Environmental Pollution Caused by Masked Musangs

**DOI:** 10.7759/cureus.53745

**Published:** 2024-02-06

**Authors:** Etaro Hashimoto, Hiroaki Satoh

**Affiliations:** 1 Family Medicine, Mito Kyodo General Hospital, Tsukuba, JPN; 2 Respiratory Medicine, Mito Medical Center, Mito, JPN

**Keywords:** human living environment, wild animals, environmental destruction, masked musang, hypersensitivity pneumonitis (hp)

## Abstract

Hypersensitivity pneumonitis is an allergic disease caused by various factors such as animal proteins and chemicals. The masked musang, a small animal of the Viverridae family native to East Asia, tends to infiltrate spaces like the attics of residences, causing damage through the deposition of excrement and other means. The older Japanese patient had been experiencing cough, shortness of breath, and fever for two months before presenting to our hospital. The symptoms improved upon admission to a local medical facility but deteriorated upon discharge. This cycle was repeated twice before the patient was admitted to our hospital. Based on the recurrent pattern of improvement during hospitalization and exacerbation upon returning home, along with the results of CT imaging and bronchoscopy, we suspected hypersensitivity pneumonitis. An environmental investigation at the patient's residence revealed a masked musang nest in the attic above the patient's room. After cleaning the attic, the symptoms did not recur. Consequently, we diagnosed hypersensitivity pneumonitis due to living environmental pollution caused by masked musangs. To the best of our knowledge, there have been no previous case reports of hypersensitivity pneumonitis caused by masked musangs. When wild animals invade human living environments, there is a possibility that not only infectious diseases but also immunological disorders, including allergic diseases, may appear.

## Introduction

The masked musang (*Paguma larvata*, masked palm civet) is a small animal belonging to the Viverridae family, native to East and Southeast Asia. Masked musangs are known to inhabit areas such as attics, under floors, and storerooms of houses, causing damage by excreting feces and urine [1]. Urine can stain attics, emit strange odors, and in some cases can have a negative impact on residents' health [2,3]. Inhaling organic dust, chemicals, microorganisms, or dust containing animal proteins (e.g., avian feathers) can cause a hypersensitivity reaction in the lungs, known as hypersensitivity pneumonitis [4-10]. To our best knowledge, there have been no case reports of hypersensitivity pneumonitis attributable to exposure to environmental pollutants from masked musang (e.g., their feces, urine, or dander). We treated a patient who was diagnosed with hypersensitivity pneumonitis, attributed to environmental pollution in the living environment caused by a masked musang residing in the attic. This report aims to provide insights into diagnostic strategies and therapeutic approaches for patients afflicted with similar conditions.

## Case presentation

**Figure 1:**
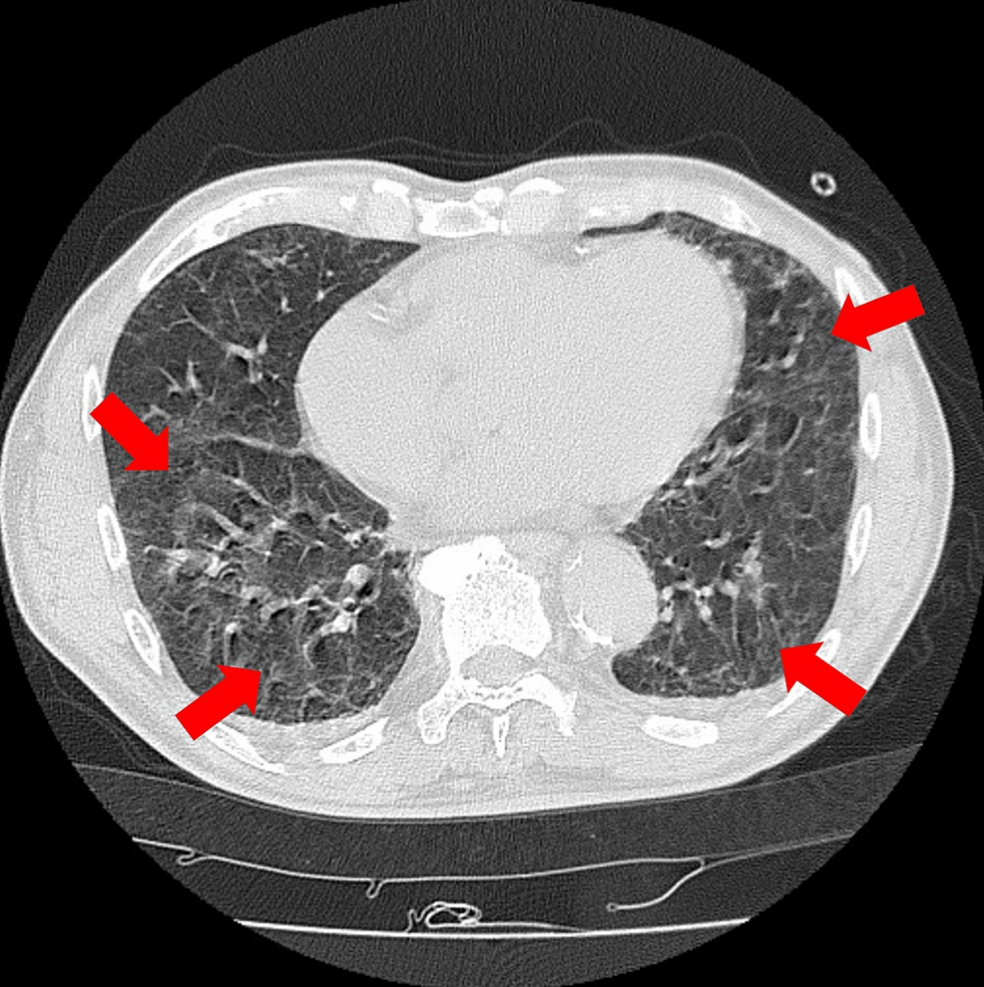
Mosaic attenuation in both lungs on a plain thoracic CT at the Th10 level (arrow)

An 81-year-old Japanese man visited a clinic two months before presenting at our hospital (early April) with symptoms of dry cough, shortness of breath, and fever. Prior to the onset of symptoms, there was no history of newly prescribed medications or respiratory infections such as upper respiratory tract infection. He was suspected of having bacterial pneumonia and was prescribed cefditoren pivoxil, which he took for five days without symptomatic improvement. Subsequently, he was prescribed clarithromycin for five days by the same clinic, yet his symptoms persisted. One month before visiting our hospital, the patient was referred to a hospital and was admitted. He was again suspected of bacterial pneumonia, and ceftriaxone was started. His symptoms improved the day after admission. After receiving ceftriaxone for seven days, he was discharged home. However, cough and shortness of breath recurred on the night of his discharge, and he was readmitted to the same hospital the following day. Ceftriaxone was administered again, and he was discharged home after 10 days without further recurrence of symptoms. The symptoms did not recur after that. However, on the morning of his visit to our hospital (late May), cough, shortness of breath, and fever reappeared, and he was transported to our hospital. The patient lived in a two-story house built 30 years ago, and the patient's room was located on the second floor. He had no history of smoking or inhalation and was living a retired life. He was living with his wife, and occasionally, children and grandchildren would come to visit. No family members or individuals in the patient's surroundings showed similar symptoms. Upon auscultation of the chest, bilateral fine crackles were audible in the base of both lungs. The arterial oxygen saturation was 88% in room air. On admission, his white blood cell count was 9400/μL (neutrophil 92.3 %, eosinophil 1.2 %) and C-reactive protein was 2.17 mg/dL. Due to the unavailability of infectious disease panel tests (as it is not widely available in Japan), it was not conducted. A chest CT scan revealed mosaic attenuation in both lung fields (Figure 1).

The cytological examination of the bronchoalveolar lavage fluid revealed neutrocytosis (neutrophil 73 and the cluster of differentiation (CD) 4/8 ratio of the fluid was low, at 1.02). The specimens obtained by transbronchial lung biopsy revealed alveolitis. 

Based on the clinical course, which involved repeated improvements during hospitalization and deteriorations upon discharge, we suspected hypersensitivity pneumonitis. We decided to observe the patient's condition without administering any drugs, providing only oxygen therapy. Initially, the patient received oxygen at a rate of 6 L/min. However, within a few hours after admission, there was a rapid improvement in the respiratory status. Six hours post-admission, the patient was able to walk without the need for supplemental oxygen. The day after admission, the symptoms disappeared. At this point, we conducted a more detailed medical history inquiry, including the patient's living environment, activities, and any known interactions with animals. Additionally, exposure tests were performed during the hospitalization, which included items such as feather duvets and pillows that the patient had purchased five months prior to admission. Furthermore, serological testing for specific antibodies (e.g., *Trichosporon asahii*) was conducted. However, none of these efforts were successful in identifying the antigen. After a trial outing to his home, during which he stayed at home from 10 am to 5 pm without a recurrence of symptoms, the patient was discharged home on the tenth day of hospitalization. However, six days later, the symptoms recurred, leading to readmission.

**Figure 2:**
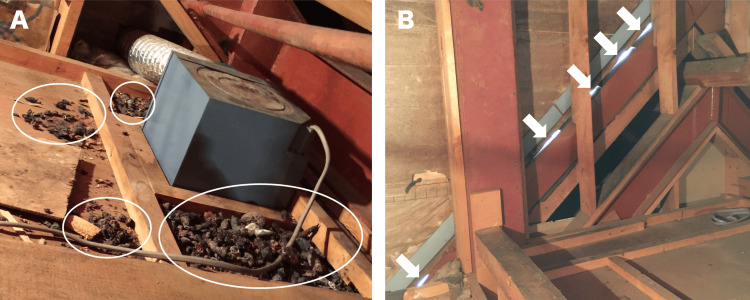
The attic of the patient's house (A) Masked musang droppings in the attic (circle). (B) Holes in the wall that connect to the outdoors (arrow).

To identify the antigen, the first author (EH) conducted an environmental investigation of the patient's residence. There were no marshes, forests, animal breeding facilities, or factories in the vicinity of the home, and no birds were observed in the early morning. The water facilities in the patient's home (kitchen, bathroom, and washbasin) and the air conditioning filters were found to be clean, with no signs of mold. He noted that the patient's room was on the top floor. Upon observing the attic space directly above the patient's room, a nest of masked musangs was discovered (Figure 2).

**Figure 3:**
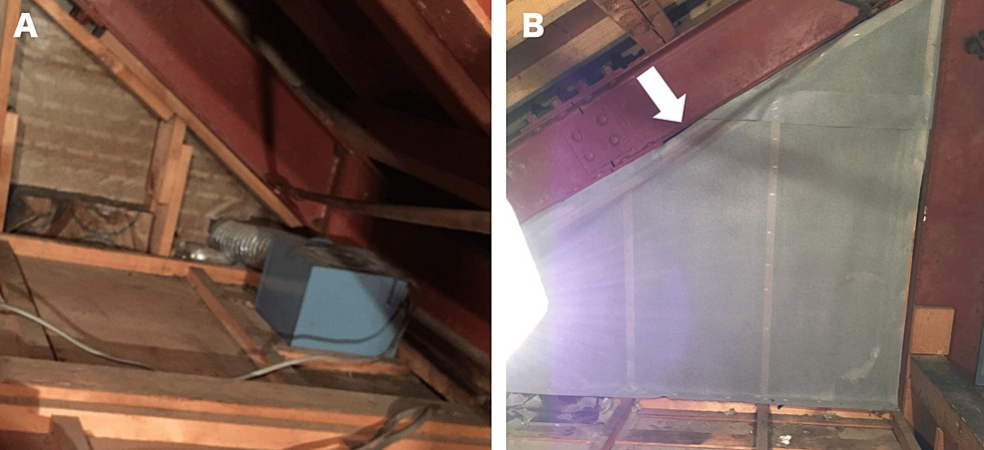
The attic after cleaning (A) The attic from which the droppings were removed. (B) The holes are covered with a sheet (arrow).

Considering the environmental pollution caused by masked musang as a potential cause of hypersensitivity pneumonitis, we requested the patient's cooperation, and a cleaning service was employed to clean and remove the nests from the attic (Figure 3).

**Figure 4:**
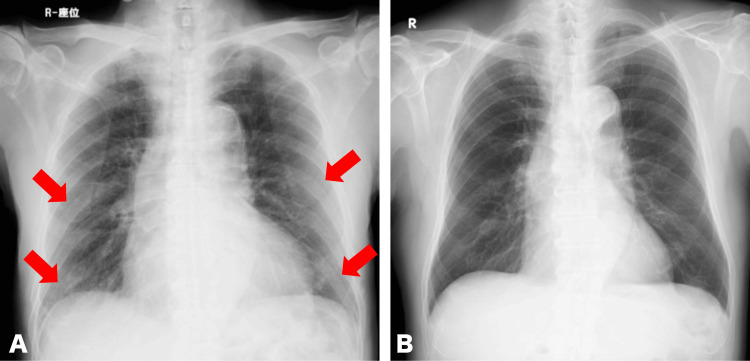
Chest X-ray (A) Ground glass opacity (arrow), at the first visit (seated position, AP view). (B) Disappeared abnormal shadows, at the follow-up visit (standing position, PA view).

After returning home, he experienced no worsening of symptoms. Subsequent imaging examinations were conducted, revealing no abnormal shadows (Figure 4).

From the above, the patient was diagnosed with hypersensitivity pneumonitis attributed to environmental pollution caused by masked musangs. After a follow-up period of six years without any treatment, no recurrence was observed.

## Discussion

Hypersensitivity pneumonitis is caused by repeated inhalation of organic or inorganic dust such as fungal spores, bacteria, and animal proteins and is characterized by type III or type IV allergic reactions [4-11]. According to international guidelines, such as those established by the American Thoracic Society, ground-glass opacities, poorly defined centrilobular nodules, mosaic attenuation on inspiratory CT images, and air trapping on expiratory CT images are typical patterns of non-fibrotic hypersensitivity pneumonitis [12]. Testing for specific antibodies against the causative antigen is effective for diagnosis [13,14]. Environmental investigations are sometimes conducted to identify specific antigens [15]. The treatment is based on the avoidance of identified antigens. In patients with severe symptoms or those with chronic conditions, steroids and immunosuppressive drugs may be used to control allergic inflammation and prevent lung fibrosis [12]. There are many reports of hypersensitivity pneumonitis due to inhalation of organic dust, chemicals, microorganisms, or dust containing avian or animal proteins [4-10]. There have been no previous case reports of hypersensitivity pneumonitis caused by masked musangs, and similar to prior reports, this case illustrates that a detailed environmental investigation is effective in diagnosing the disease. Had the environmental investigation not identified the cause, the symptoms might have persisted and worsened, potentially leading to unnecessary treatments such as steroid administration.

Environmental destruction caused by humans is narrowing the areas in which wild animals can survive [16]. On the other hand, there are quite a few species of wild animals that have adapted well to the environment in which humans live and have even expanded their range of living areas [17]. These changes may lead to the emergence of new diseases and conditions that were previously inconceivable [18,19]. This patient also had these backgrounds, and it was supposed that a previously unthinkable pathological condition had appeared. Masked musangs often gather glass wool, a commonly used building material, in their living environments. In addition, they have gathered materials from plants and animals to create habitats [18]. It is presumed that this wild animal living in the attic deteriorates the sanitary environment, including excrement. In these circumstances, it was not possible to determine what was the inflammatory substance that caused the onset of hypersensitivity pneumonitis. There were numerous substances of concern, including animal proteins and fungi found in the feces, urine, fur, and dander of the masked musang. However, challenge tests for each of these substances were impractical, necessitating the deferral of antigen identification to future research.

The COVID-19 pandemic has heightened awareness of the escalating human impact on the natural environment. Simultaneously, the adaptation and coexistence of wild animals in human habitats are identified as potential sources of new disease outbreaks [20]. There is a possibility that the number of wild animals, not just masked musangs, that increase their numbers by approaching human living spaces is likely to increase. For patients presenting with recurring or persistent respiratory symptoms, it is necessary to consider hypersensitivity pneumonitis in the differential diagnosis along with infectious diseases. If hypersensitivity pneumonitis is suspected and the antigen cannot be identified through general inquiry and tests, a detailed environmental investigation considering such wildlife is required.

## Conclusions

When wild animals intrude into human living environments, not only infectious diseases but also immunological disorders, including allergic diseases, may occur. Although still rare, healthcare professionals should consider hypersensitivity pneumonitis due to environmental pollution by wild animals when evaluating patients with persistent respiratory symptoms. To identify the antigens of hypersensitivity pneumonitis, a detailed environmental investigation, in addition to hospital inquiries and tests, is effective.
